# Perceived societal meritocratic climate and self-stigma among adolescents in conflict with the law: the mediating role of collective self-esteem and social disconnection

**DOI:** 10.3389/fpsyg.2026.1852343

**Published:** 2026-09-15

**Authors:** Xiaoyi Wang, Wanlu Li, Rong Liu, Xue Zhang, Weijie Meng

**Affiliations:** 1 College of Education, Ludong University, Yantai, China; 2 Institute for Education and Treatment of Problematic Youth, Ludong University, Yantai, China; 3 Shandong Business Institute, Yantai, China; 4 Weifang No. 1 Middle School, Weifang, China

**Keywords:** perceived societal meritocratic climate, self-stigma, adolescents in conflict with the law, social disconnection, collective self-esteem

## Abstract

**Introduction:**

This research investigated the relationship between perceived meritocratic climate and self-stigma among adolescents in conflict with the law across two studies.

**Methods:**

Study 1 (*N* = 322) employed a cross-sectional survey; Study 2 (*N* = 254) used an experimental manipulation of perceived (non)meritocratic climate.

**Results:**

In Study 1, perceived meritocratic climate positively predicted self-stigma, and an exploratory serial mediation via collective self-esteem and social disconnection was observed. In Study 2, the serial mediation was not replicated; instead, the effect was mediated solely by social disconnection.

**Discussion:**

These findings suggest that perceived meritocratic climate, as a subjective manifestation of structural social evaluation, reinforces a dual stigmatized identity of being both an offender and a failure. Short-term interventions should focus on enhancing social connectedness to buffer against self-stigma, while long-term efforts should facilitate integration into socially valued and accepting groups, thereby elevating collective self-esteem, strengthening social connection, and ultimately reducing self-stigma.

## Background

In China, specialized schools serve as official institutions for the education and correction of juvenile delinquents, admitting adolescents aged 12–18 with severe problem behaviors or those exempted from criminal punishment due to age (Yao and Liu, 2024; Yao and Shen, 2025). Although intended for protective education, these schools objectively incorporate punitive elements, including liberty restrictions (Liu, 2024). Consequently, admission imposes a stigmatizing label, which, especially for repeat offenders, often crystallizes into profound self-stigma.

However, delinquency-based self-stigma is not purely individual but is embedded within the broader sociocultural climate. In China, meritocracy—legitimized by narratives such as “hard work leads to success” and “education changes destiny”—fosters differential evaluations of “winners” and “losers.” Its dark side is increasingly evident, as it rationalizes inequality, fuels group antagonism, and devalues individuals (Kuppens et al., 2018; Tomova Shakur and Phillips, 2022). Extant research has largely treated meritocracy as a macro-level distributive principle, overlooking its psychologically proximal impact as a pervasive perceived climate. Does this climate impose additional psychological strain and self-deprecation on vulnerable groups at the bottom of the meritocratic hierarchy? To address this gap, the present study focuses on juvenile offenders and examines the mechanisms through which perceived societal meritocratic climate shapes their self-stigma.

### Understanding perceived societal meritocratic climate

Meritocracy is commonly understood as a social system in which wealth, power, status, and opportunities are allocated according to individual effort, talent, and contribution (Littler, 2018). It embodies an appealing promise: those who work hard and demonstrate ability can rise, regardless of background. Yet the term was originally coined as satire by Young (1994), and its contemporary celebration often obscures a darker reality—rather than fostering genuine equity, meritocracy can legitimize inequality and erode public sympathy for disadvantaged groups (Sandel, 2020; García-Sierra, 2023).

Empirical research has struggled to capture this complexity. Studies have often relied on proxy constructs such as Protestant work ethic, belief in a just world, and status legitimacy (Rüsch et al., 2010; Day and Fiske, 2017; Laurin et al., 2011), resulting in considerable conceptual overlap (Gilbert, 2023). Son Hing et al. (2011) distinguished descriptive meritocracy—whether society actually allocates resources based on merit—from prescriptive meritocracy—the normative ideal that it should. However, as Madeira et al. (2019) noted in their systematic review, existing measures remain conceptually vague and often fail to differentiate between these two forms. To address this, Hoyt et al. (2021) integrated individual dimensions (effort, internal control) with structural dimensions (social mobility, equal opportunity) to capture meritocracy as a multidimensional construct. Yet these approaches essentially assess people’s meritocratic beliefs and do not directly capture what Sandel (2020) termed the “tyranny of merit”—the separation of social groups based on merit, the attribution of outcomes solely to individual effort and ability, and society’s differential treatment of winners and losers—thereby failing to tap individuals’ felt experience of this merit-based logic and its consequences.

To address these ambiguities, we introduce the construct of perceived societal meritocratic climate, defined as an individual’s ambient perception that society evaluates, judges, and attributes success and failure to personal merit. This concept foregrounds the lived experience of being evaluated through a meritocratic lens, rather than a factual judgment (descriptive), a normative preference (prescriptive), or a generalized justice belief. It directly captures the “tyranny of merit” dynamic. To clarify the construct’s extension, Table 1 illustrates how high versus low levels of this perceived climate manifest, and Table 2 delineates its conceptual boundaries with related constructs, along with their potential psychological implications for disadvantaged groups.

**TABLE 1:** Manifestations of high vs. low perceived social meritocratic climate.

Level	Evaluative climate dimension	Attributional tendency dimension	Psychological experience of disadvantaged groups
High	Strongly perceives society’s differential treatment of winners and losers	Strongly perceives that society attributes success/failure entirely to personal responsibility	Threat, pressure, lack of dignity
Low	Less aware of differential treatment based on merit criteria	Believes that society acknowledges the role of uncontrollable factors (e.g., luck, family background) in shaping outcomes	Relatively at ease, less troubled by evaluative pressure

**TABLE 2:** Conceptual boundaries among related constructs.

Concept	Core question	Focus of attention	Socio-psychological function for disadvantaged groups
Perceived social meritocratic climate	Subjective perception of merit-based evaluation	How society differentiates between winners and losers	Perception of pressure and self-blame
Descriptive meritocracy (Son Hing et al., 2011)	Factual judgment about whether meritocracy operates in society	Whether resources are distributed according to ability/effort	Sense of control or fairness
Prescriptive meritocracy (Son Hing et al., 2011)	Value claim about whether meritocracy should operate	Whether resources ought to be distributed according to ability/effort	Ideal reference
Belief in a just world (Dalbert, 1999)	Belief in the fairness of the world	Whether good/bad deeds receive corresponding outcomes	System justification
Protestant work ethic (Quinn and Crocker, 1999)	Endorsement of the virtue of diligence	Effort and individual responsibility	Self-discipline

Thus, perceived societal meritocratic climate captures the lived experience of being evaluated, judged, and positioned within a social order governed by meritocratic logic, rather than reflecting descriptive beliefs, prescriptive ideals, or general justice beliefs. Delinquent adolescents may simultaneously endorse meritocratic attributions—that success and failure stem from personal effort and ability—while keenly sensing society’s veneration of winners and contempt for losers, a tension that reveals the very complexity of the meritocracy concept.

### Meritocracy and its detrimental effects on disadvantaged groups

Meritocracy belief can offer disadvantaged groups a sense of hope and personal control (McCoy et al., 2013; Rüsch et al., 2010; Morris et al., 2022). when meritocratic beliefs persistently clash with harsh realities, they often trigger frustration and collapse of self-efficacy, leaving individuals more deeply entrenched in disadvantage (García-Sierra, 2023). Moreover, in systems that are not truly meritocratic, low-status groups tend to internalize failure as personal responsibility, thereby legitimizing the system, while high-status groups blame the disadvantaged as “undeserving”. This myth—that success depends solely on effort—erodes humility, the common good, and social solidarity, inflicting particular harm on the most vulnerable (Andre, 2025; Darnon et al., 2018; Trevisan et al., 2022).

On one hand, meritocracy weakens public empathy and reduces support for institutional remedies. Its logic attributes outcomes to individual ability and effort, making disadvantage appear as a matter of “incompetence” or “insufficient effort”, which invites moral condemnation and stigma. The less educated are often perceived as lazy and incapable (Kuppens et al., 2018); the unemployed as unreliable and untrustworthy (Rauscher et al., 2024); and the poor face greater blame and less sympathy (García-Sierra, 2023; Koo et al., 2023). Those who endorse meritocratic beliefs are less supportive of preferential policies for the disadvantaged (Son Hing et al., 2002) and may even perceive discriminatory allocations as fair (Tomova Shakur and Phillips, 2022). This outcome-oriented attribution systematically ignores structural disparities in family capital, education, and social class, leaving resource distribution and responsibility judgments unchanged even when initial disadvantages are recognized (Andre, 2025). In essence, meritocracy functions as a punitive mechanism: disadvantaged groups are not only excluded from resources but also deprived of social dignity under its yardstick.

On the other hand, low-status group members internalize meritocratic attributions, transforming external devaluation into self-blame and self-stigma. They are disciplined by a logic of “deserved failure”—a core element of what Sandel (2020) terms the “tyranny of merit,” which strips people of both material resources and social dignity, thereby turning a seemingly fair ideology into systemic discrimination (Bohren et al., 2025). For example, lower educational attainment is attributed to personal effort, and those affected bear moral stigma and come to view their plight as deserved (Kuppens et al., 2018). Similarly, individuals with obesity who believe weight is self-controllable become more accepting of weight stigma (Tomova Shakur and Phillips, 2022). Overall, meritocracy entangles everyone in a logic that promises fair returns while ignoring initial inequalities in talent and resources, ultimately imposing moral burdens on those who fail (Littler, 2018).

### Linking perceived societal meritocratic climate to self-stigma among adolescents in conflict with the law

In China, meritocracy, combined with a deeply entrenched punitive orientation, has jointly shaped the public stereotype that juvenile offenders are “deserving of their fate” and “unworthy of sympathy.” In reality, these adolescents commonly face structural disadvantages—family dysfunction, inadequate educational support, and institutional barriers to post-release employment (Di, 2012; Huang, 2019; Bai and You, 2025). Under the screening logic of meritocracy, such systemic disadvantages are reinterpreted as evidence of insufficient personal effort. This intensifies external discrimination and generates a strong sense of relative deprivation; when the link between hard work and a decent life is broken, some adolescents turn to illegal means to obtain alternative “success,” thereby repeatedly reinforcing a deviant identity. The resulting public rejection and denigration are gradually internalized through sustained social interaction, leading to self-stigma.

Although no research has directly linked perceived societal meritocratic climate to self-stigma in delinquent populations, existing studies offer important clues. Meritocratic beliefs can mask explicit self-stigma in individuals with mental illness while fostering deeper implicit guilt and self-blame (Rüsch et al., 2010). In school settings, a stronger meritocratic climate increases the risk of bullying among academically disadvantaged adolescents (Pan and Li, 2026). Moreover, stronger descriptive meritocratic beliefs are associated with harsher rejection of illegal behaviors, while weaker meritocratic environments correspond to greater tolerance (Koo et al., 2024). However, most studies have focused on advantaged groups or the general public and seldom examine the impact on in-group attitudes from the subjective perspective of low-status groups. The present study thus introduces perceived societal meritocratic climate to uncover the overlooked consequences of meritocracy for the disadvantaged.

*H1*: Perceived societal meritocratic climate positively predicts self-stigma among juvenile offenders. Exploratorily, we further propose that a perceived non-meritocratic climate may reduce their self-stigma.

### The chain mediating role of collective self-esteem and social disconnection

Social identity theory posits that self-concept is constructed from both personal and social identities, with social identity arising from identification with one’s group (Hogg and Abrams, 2011; Ellemers et al., 2002). Drawing on the identity threat model of stigma (Major and O’Brien, 2005), we argue that perceived societal meritocratic climate may serve as a stigma-relevant threat cue for low-merit groups, exacerbating self-stigma through the combined pathways of damaged ingroup collective self-esteem and obstructed social connectedness with the broader society. Collective self-esteem—the individual’s perception of how their group is evaluated by others—constitutes an important source of self-worth (Luhtanen and Crocker, 1992; Lyu et al., 2017), while social disconnection refers to a sense of alienation, isolation, and emotional detachment experienced in interpersonal, group, and societal relationships (Wu et al., 2020). According to need-to-belong theory, social exclusion thwarts the fundamental need to belong, leading to diminished social connectedness (Baumeister and Leary, 1995), and lowered collective self-esteem has been shown to weaken the motivation to form positive emotional bonds with the broader society.

In a meritocracy-oriented cultural context, visible merit serves as the key yardstick for distinguishing winners from losers (Wu and Gao, 2023). Juvenile offenders, marked by low socioeconomic status, limited educational attainment, and a conspicuous deviant label, are readily categorized as “incompetent” or “morally tainted” failures, incurring multiple layers of identity threat. A strong societal meritocratic climate further consolidates the intergroup boundary between them and mainstream groups, weakening their willingness to integrate. The social disconnection arising from impaired collective self-esteem is often accompanied by heightened negative affect, reduced self-regulation, and social withdrawal, rendering them more susceptible to internalizing external devaluation as self-stigma (Baumeister et al., 2007). Moreover, the deindividuation effect of a stigmatized identity can prompt these adolescents to adopt attitudes and behaviors consistent with the deviant stereotype, reinforcing delinquent patterns and impeding positive identity reconstruction (Jetten et al., 2015).

Empirical evidence indicates that positive collective self-esteem buffers self-stigma and enhances well-being (Longares et al., 2016), whereas social disconnection is negatively correlated with multiple dimensions of collective self-esteem (Fan et al., 2015). These findings suggest that declining collective self-esteem and rising social disconnection may not only reinforce each other but also jointly mediate the pathway from perceived societal meritocratic climate to self-stigma. Building on this reasoning, we propose:

***H2:*** Collective self-esteem and social disconnection play a chain mediating role between perceived societal meritocratic climate and self-stigma among juvenile offenders. Specifically, perceived societal meritocratic climate reduces collective self-esteem, which in turn heightens social disconnection, ultimately exacerbating self-stigma; each variable may also function as an independent mediator. The proposed theoretical model is illustrated in Figure 1.

**FIGURE 1:**
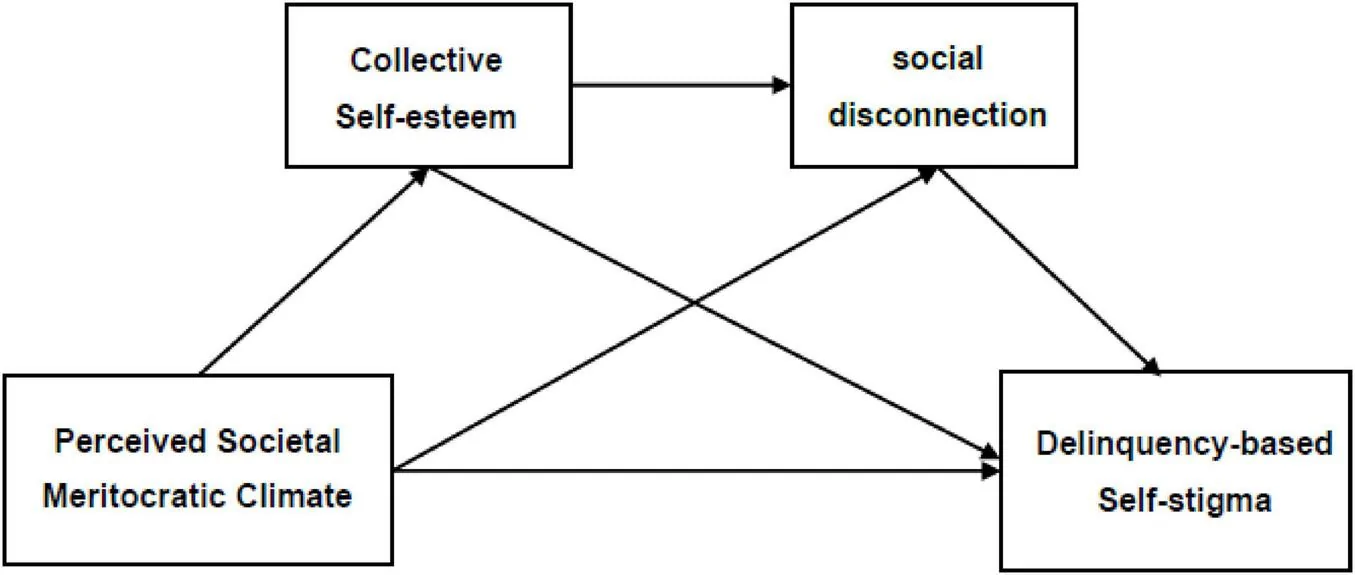
Theoretical model.

## Study 1

Study 1 uses a cross-sectional survey to examine the relationships among perceived societal meritocratic climate, collective self-esteem, social disconnection, and self-stigma, providing an initial test of the proposed theoretical model.

### Methods

#### Ethical statement

This study was approved by the Institutional Review Board of Lu Dong University (Approval No. LDU-IRB2026041003) on April 10, 2026. Informed consent was obtained from all participants.

#### Participants

A cluster sampling method was used to recruit participants from five specialized schools in Province G. A total of 400 questionnaires were distributed, and after excluding participants who failed attention checks, 322 valid responses were retained (253 male; *M*_*age*_ = 14.76, *SD*_*age*_ = 1.19). A sensitivity analysis (α = 0.05, *power* = 0.80) based on the regression model with the most predictors (8 predictors) indicated that the minimum detectable effect size was *f*^2^ = 0.048 (corresponding to *R*^2^ = 0.046), The observed *R*^2^ confirm that the sample size was adequate for robust parameter estimation. All indirect effects were tested using bias-corrected bootstrap confidence intervals with 5,000 resamples to ensure inferential robustness.

#### Procedure and measures

A After providing informed consent, participants completed measures of perceived societal meritocratic climate, collective self-esteem, social disconnection, delinquency-based self-stigma, and demographics. Because the serial mediation pathway was introduced during revision based on theoretical considerations rather than pre-registered a priori, all analyses involving the collective self-esteem → social disconnection chain are reported as exploratory.

#### Perceived societal meritocratic climate

This self-developed scale consists of 15 items forming two dimensions: Perceived Meritocratic Evaluation Climate (10 items; e.g., “In society, losers are easily looked down upon or belittled.”) and Perceived Meritocratic Attribution Tendency (5 items; e.g., “Most failures in life are due to one’s own lack of effort or laziness.”). Items were rated on a 6-point scale (1 = *strongly disagree*, 6 = *strongly agree*), with higher total scores indicating a stronger perceived meritocratic climate. Confirmatory factor analysis in the present sample showed good model fit (χ^2^/*df* = 1.88, *RMSEA* = 0.058, *CFI* = 0.934, *TLI* = 0.911, *SRMR* = 0.053). *Cronbach’s* α = 0.863.

#### Self-stigma scale for adolescents in conflict with the law

This self-developed 15-item scale comprises two dimensions: Internalized Stigma (7items; e.g., “I feel inferior due to my offending identity.”) and Perceived Stigma (8 items; e.g., “Most people look down on delinquent adolescents like me.”). Items were rated on a 6-point scale (1 = *strongly disagree*, 6 = *strongly agree*), with higher scores reflecting greater self-stigma. *Cronbach’s* α = 0.915.

#### Collective self-esteem scale

Collective self-esteem was assessed using the Chinese version of the Collective Self-Esteem Scale (Luhtanen and Crocker, 1992; revised by Zhang and Liang, 2002). The scale comprises 8 reverse-scored items rated on a 7-point scale (1 = *strongly disagree*, 7 = *strongly agree*), with higher scores indicating higher collective self-esteem as a member of the juvenile offender group. *Cronbach’s* α = 0.785.

#### Social disconnection scale

The 10-item Social Disconnection subscale of the Chinese Social Connectedness Scale (Lee et al., 2001; revised by Fan et al., 2015) was used. Items were rated on a 6-point scale (1 = *strongly disagree*, 6 = *strongly agree*), with higher scores indicating lower social connectedness with the normative group. *Cronbach’s* α = 0.878.

#### Data analysis

Data analysis was performed using SPSS 26.0. Participants who failed the attention check were excluded, and missing values were handled using multiple imputation. Common method bias was assessed using Harman’s single-factor test. Descriptive statistics and Pearson correlations were calculated for the main variables. Multicollinearity was examined by computing variance inflation factors (VIFs). The serial mediation model was tested using the PROCESS macro (Model 6) for SPSS. Indirect effects were evaluated using bias-corrected bootstrap 95% confidence intervals based on 5,000 resamples.

## Results

### Test for common method bias

Because all variables were measured via self-report, common method bias was assessed using Harman’s single-factor test (Zhou and Long, 2004). The unrotated factor solution yielded 22 factors with eigenvalues greater than 1, and the first factor accounted for 15.27% of the total variance, well below the 40% threshold, indicating that common method bias was not a serious concern.

### Descriptive statistics and correlation analysis

Table 3 presents the descriptive statistics and bivariate correlations among the study variables. Perceived societal meritocratic climate was positively correlated with self-stigma (*r* = 0.537, *p* < 0.01), providing preliminary support for Hypothesis 1. It was also positively associated with social disconnection (*r* = 0.301, *p* < 0.01) and negatively associated with collective self-esteem (*r* = -0.159, *p* < 0.01). Social disconnection was negatively correlated with collective self-esteem (*r* = -0.471, *p* < 0.01) and positively correlated with self-stigma (*r* = 0.515, *p* < 0.01). Self-stigma was negatively correlated with collective self-esteem (*r* = -0.303, *p* < 0.01). Overall, higher perceived societal meritocratic climate was associated with lower collective self-esteem, stronger social disconnection, and greater self-stigma among juvenile offenders.

**TABLE 3:** Descriptive statistics and correlations among variables (*n* = 322).

Variables	*M*	*SD*	1	2	3
1 Perceived societal meritocratic climate	3.92	0.76	–	–0.471**	
2 Collective self-esteem	4.21	0.62	–0.159**		
3 Social disconnection	3.14	0.93	0.301**		
4 Self-stigma	3.21	0.86	0.537**	–0.303**	0.515**

**p < 0.01.

### The chain mediating role of collective self-esteem and social disconnection

To examine the mediating mechanisms, we tested a serial mediation model (PROCESS Model 6; Hayes, 2013) with perceived societal meritocratic climate as the predictor, collective self-esteem and social disconnection as serial mediators, and self-stigma as the outcome, controlling for gender, age, and offense type. All variance inflation factors were below 2.00, indicating no serious multicollinearity. Indirect effects were evaluated using bias-corrected bootstrap 95% confidence intervals based on 5,000 resamples. We applied Benjamini–Hochberg FDR correction (*q* < 0.05) for multiple testing; reported p-values are FDR-adjusted.

As shown in Table 4, perceived societal meritocratic climate negatively predicted collective self-esteem (β = -0.145, *p* < 0.01) and positively predicted social disconnection (β = 0.213, *p* < 0.001). Collective self-esteem negatively predicted social disconnection (β = -0.416, *p* < 0.001). In the full model predicting self-stigma, both perceived societal meritocratic climate (β = 0.414, *p* < 0.001) and social disconnection (β = 0.349, *p* < 0.001) were significant positive predictors, whereas collective self-esteem was not (β = -0.072, *p* > 0.05). The path model is presented in Figure 2.

**TABLE 4:** Regression results for the serial mediation model (*n* = 322).

Outcome variable	Predictor variable	*R*	*R2*	*F*	β	*t*
Collective self-esteem		0.249	0.062	1.864*		
	Perceived societal meritocratic climate				–0.145	–2.577**
Social disconnection		0.574	0.330	12.709***		
	Perceived societal meritocratic climate				0.213	4.421***
	Collective self-esteem				–0.416	–8.674***
Delinquency-based self-stigma		0.674	0.454	19.764***		
	Perceived societal meritocratic climate				0.414	9.219***
	Collective self-esteem				–0.072	–1.477
	Social disconnection				0.349	6.794***

*p < 0.05,

**p < 0.01,

***p < 0.001. All *p*-values are FDR-adjusted at *q* < 0.05.

**FIGURE 2:**
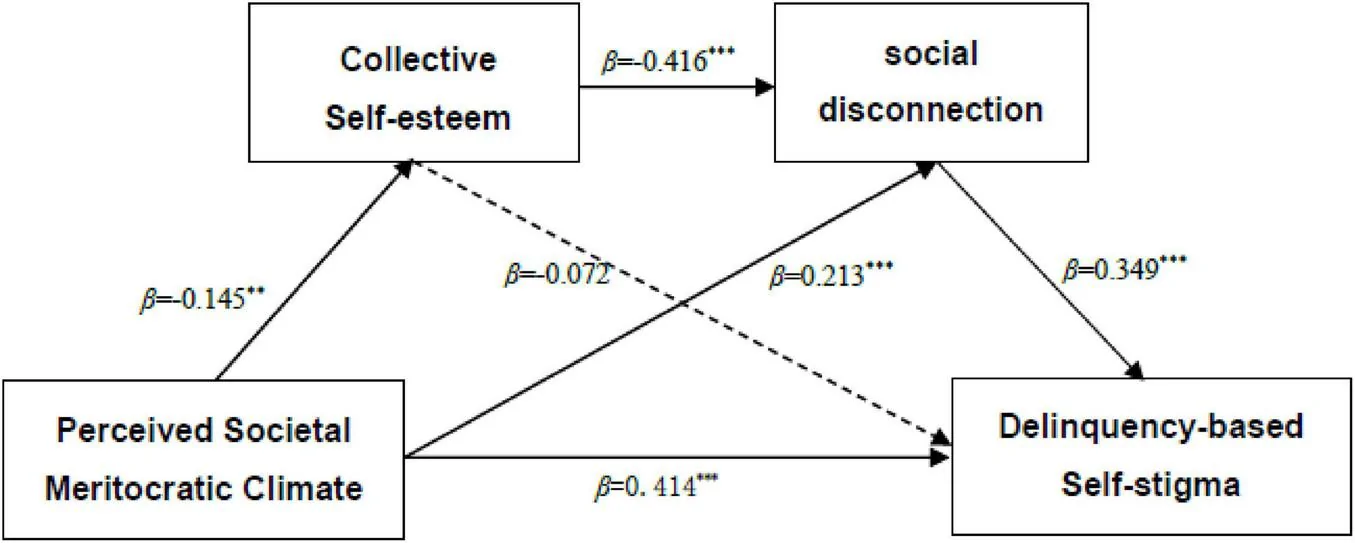
Serial mediation model of collective self-esteem and social disconnection in the relationship between perceived societal meritocratic climate and self-stigma among justice-involved adolescents. **p < 0.01, ***p < 0.001.

Mediation effect (Table 5) indicated that the total effect of perceived societal meritocratic climate on self-stigma was significant (*b* = 0.586, 95%CI [0.480, 0.692]), as was the direct effect (*b* = 0.467, 95%CI [0.367, 0.566]), accounting for 79.69% of the total effect. The total indirect effect was also significant (*b* = 0.119, 95%CI [0.066, 0.182]), representing 20.31% of the total effect. Specifically, the path through social disconnection alone was significant (*b* = 0.084, 95%CI [0.042, 0.132]), accounting for 70.59% of the total indirect effect. The serial mediation path via collective self-esteem and social disconnection was significant as well (*b* = 0.024, 95%CI [0.003, 0.048]), explaining 20.17% of the total indirect effect. The remaining path through collective self-esteem alone was not significant (*b* = 0.017, 95%CI [–0.006, 0.038]). Thus, the association between perceived societal meritocratic climate and self-stigma was partially mediated by social disconnection and serially mediated by collective self-esteem and social disconnection, with social disconnection emerging as the primary mediator.

**TABLE 5:** Mediation effect test.

Effect type and path	Effect (unstandardized coefficient *b*)	Percentage	95% CI
Total effect decomposition		(of total effect)	
Total effect	0.586	100%	[0.480, 0.692]
Direct effect	0.467	79.69%	[0.367, 0.566]
Total indirect effect	0.119	20.31%	[0.066, 0.182]
Indirect path decomposition		(of indirect effect)	
Path1	0.017	14.29%	[-0.006, 0.038]
Path2	0.084	70.59%	[0.042, 0.132]
Path 3	0.024	20.17%	[0.003, 0.048]

Path 1 = Perceived meritocratic climate → Collective self-esteem → Self-stigma; Path 2 = Perceived meritocratic climate → Social disconnection → Self-stigma; Path 3 = Perceived meritocratic climate → Collective self-esteem → Social disconnection → Self-stigma.

### Supplementary dimension-specific analyses

To examine whether the observed effects were driven by both dimensions of perceived societal meritocratic climate or primarily by one, we reran the same serial mediation model separately for the evaluative climate dimension and the attributional tendency dimension, controlling for the same covariates (see Part 4 of the Supplementary material). Both dimensions were positively associated with self-stigma, and in both models social disconnection significantly mediated the relationship between the perceived climate dimension and self-stigma. The exploratory serial pathway via collective self-esteem and social disconnection was also significant for both dimensions, although the effect sizes were small; for the evaluative climate dimension, the confidence interval only narrowly excluded zero, suggesting a marginal effect. Standardized estimates indicated that the attributional tendency dimension may be somewhat more strongly associated with self-stigma than the evaluative climate dimension. These supplementary results suggest that the total-score effect is not driven solely by one dimension; rather, both dimensions contribute, with social disconnection serving as the common primary mediator.

### Discussion

These results provide preliminary support for Hypothesis 1 and for the mediating role of social disconnection (H2). An exploratory serial mediation analysis suggested that perceived meritocratic climate was indirectly associated with self-stigma through lower collective self-esteem and subsequently greater social disconnection. However, because this serial pathway was specified *post-hoc* and based on cross-sectional data, it should be interpreted with caution and requires confirmatory evidence.

Collective self-esteem did not independently mediate the link; its effect was transmitted entirely through social disconnection. One interpretation is that, under a meritocratic evaluative framework, these adolescents tend to hold relatively low ingroup evaluations, which may increase their perceived difficulty in connecting with the normative group, ultimately exacerbating self-stigma. Thus, fostering positive group identities and enhancing social connectedness could be important for reducing the internalization of stigma.

Taken together, these cross-sectional findings offer an initial mapping of the psychological pathways from perceived societal meritocratic climate to self-stigma, with social disconnection playing a central role. However, causal inferences cannot be drawn. The observed associations may also reflect reverse or reciprocal effects, and unmeasured confounders could contribute to the pattern. Experimental manipulation is needed in future research to verify the proposed causal directions.

## Study 2

Study 2 aimed to address the causal limitations of cross-sectional design in Study 1. Using an experimental manipulation, we examined the effects of perceived societal climate (meritocratic, non-meritocratic, and control) on self-stigma among justice-involved adolescents, and tested whether collective self-esteem and social disconnection serially mediated these effects. By identifying the key mediating pathways, we sought to pinpoint potential intervention targets for reducing self-stigma in this population.

### Methods

#### Participants

An a priori power analysis (G*Power 3.1; Faul et al., 2009) for a one-way ANOVA (medium effect *f* = 0.25, α = 0.05, *power* = 0.80.) indicated a minimum of 158 participants. To allow for data cleaning, we recruited 254 justice-involved adolescents; all provided informed consent before the experiment. After exclusions, the final sample comprised 254 participants (*M*_*age*_ = 14.70, *SD* = 1.05; 14.90% female). A sensitivity analysis showed that with *N* = 254, the study could detect a minimum effect *f*^2^ = 0.20 (medium) for between-group comparisons and *f*^2^ = 0.038 (small) for the multiple regression testing the serial mediation model. Thus, the sample was adequately powered to detect small-to-medium effects and above, although detecting more subtle effects would require larger samples.

#### Research design and procedure

This study used a one-factor between-subjects design with three conditions: meritocratic climate, non-meritocratic climate, and control. Participants were randomly assigned to conditions (meritocratic climate: *n* = 85; non-meritocratic climate: *n* = 84; control: *n* = 85). To enhance realism and ecological validity, the experiment was administered in small groups of 6–8 participants in a separate, dedicated space.

In the priming phase, participants read a text corresponding to their assigned condition, summarized its main idea, and provided a personal or witnessed example supporting the viewpoint to strengthen the manipulation. In the subsequent measurement phase, they completed, in order, a manipulation check, collective self-esteem, social disconnection, self-stigma, and demographic measures.

### Materials and measures

#### Priming materials and manipulation check

Priming texts were adapted from prior research (Koo et al., 2024; Gilbert, 2023) and presented as debater’s statements. The meritocratic text emphasized that success (educational attainment, occupation, wealth) sorts individuals into “winners” and “losers” based on effort. The non-meritocratic text stressed structural constraints on success and a societal shift toward diverse life values and personal meaning. The control text was a neutral nature passage. Full texts appear in Supplementary material.

The manipulation check comprised condition-specific items rated on a 6-point scale. In the meritocratic condition, items assessed perceived meritocratic evaluation and responsibility attribution (e.g., “In society, people without academic degrees are more likely to suffer discrimination”). In the non-meritocratic condition, items assessed recognition of external constraints and value diversity (e.g., “People are gradually recognizing diverse values and lifestyles”). The control group completed all items. As an indirect check, all participants rated anticipated social exclusion toward justice-involved adolescents on a 9-point scale (e.g., “In this social climate, how do you expect people to treat justice-involved adolescents like you?” 1 = *almost no exclusion*, 9 = *extreme exclusion*). Thus, the manipulation check combined direct conceptual assessment with an indirect behavioral expectation measure.

#### Collective self-esteem scale

A six-item short form of the Collective Self-Esteem Scale (Luhtanen and Crocker, 1992; Chinese translation by Zhang and Liang, 2002) was used, tapping core evaluations of ingroup identity (e.g., items 3, 7, 10, 11, 14, 15; three reverse-scored). Items were rated on a 7-point scale (1 = *strongly disagree*, 7 = *strongly agree*), with higher scores indicating higher collective self-esteem. *Cronbach’s* α = 0.731.

#### Social disconnection scale

The 10-item Social Disconnection subscale of the Chinese Social Connectedness Scale (Lee et al., 2001; revised by Fan et al., 2015) was administered. Items were rated on a 6-point scale (1 = *strongly disagree*, 6 = *strongly agree*), with higher scores indicating lower perceived positive social interactions with the normative group. Cronbach’s α = 0.863.

#### Self-stigma scale for adolescents in conflict with the law

An 8-item short form of the self-developed Self-stigma Scale for Adolescents in Conflict with the Law (SSS-ACL) was used, selecting items with high factor loadings from the perceived stigma and internalized stigma dimensions. Sample items “In this social climate, most people would look down on justice-involved adolescents like me” and “In this social climate, I feel inferior because of my delinquent status”. Items were rated on a 6-point scale (1 = *completely disagree*, 6 = *completely agree*), with higher scores indicating greater self-stigma. *Cronbach’s* α = 0.796.

#### Data analysis

Manipulation checks and group differences were first examined using one-way ANOVA in SPSS. The serial mediation model was then tested with the lavaan package in R, using bias-corrected bootstrap confidence intervals (5,000 resamples). Missing data were handled via multiple imputation.

### Results

#### Manipulation check

The priming manipulation was effective. The meritocratic group scored higher on the meritocratic perception items than the control group (*M* = 4.38, *SD* = 0.89 vs. *M* = 3.46, *SD* = 0.85), *t* (168) = 6.89, *p* < 0.001, *d* = 0.87; the non-meritocratic group scored higher on the non-meritocratic items than control group (*M* = 5.00, *SD* = 0.66 vs. *M* = 4.22, *SD* = 0.79) *t*(167) = 6.99, *p* < 0.001, *d* = 0.73. A one-way ANOVA on anticipated exclusion revealed a significant main effect, *F*(2, 251) = 67.33, *p* < 0.001. *Post-hoc* comparisons (LSD) indicated that the meritocratic group anticipated greater exclusion (*M* = 6.41, *SD* = 2.00) than both control group (*M* = 4.22, *SD* = 1.29) and non-meritocratic group (*M* = 3.33, *SD* = 1.39; all *p* < 0.001).

#### Effects of condition on the main variables

Table 6 presents descriptive statistics and one-way ANOVA results. For collective self-esteem, a significant group effect emerged, *F*(2, 251) = 6.68, *p* < 0.001, η^2^ = 0.051. The non-meritocratic group (*M* = 4.11, *SD* = 0.67) and the control group (*M* = 3.96, *SD* = 0.60), and the control group both reported higher collective self-esteem than the meritocratic group (*M* = 3.72, *SD* = 0.81, *p* < 0.01 and *p* < 0.05, respectively), while the non-meritocratic and control groups did not differ.

**TABLE 6:** Means, standard deviations, and group comparisons on outcome variables (*n* = 254).

Variable	Group	*M*	*SD*	*F*	Effect size
Collective self-esteem	Meritocratic	3.72	0.81	6.68**	η^2^ = 0.051
	Non-meritocratic	4.11	0.67		
	Control	3.96	0.60		
Social disconnection	Meritocratic	3.29	1.01	6.37**	ω^2^≈0.055
	Non-meritocratic	2.81	0.75		
	Control	2.94	0.78		
Self-stigma	Meritocratic	4.03	0.84	40.98***	η^2^ = 0.246
	Non-meritocratic	2.92	0.74		
	Control	3.35	0.82		

**p < 0.01,

***p < 0.001. All group-comparison p-values are FDR-adjusted at *q* < 0.05.

For social disconnection, Levene’s test indicated variance heterogeneity; therefore, Welch’s ANOVA and Games-Howell *post-hoc* tests were used. A significant group effect was found, Welch’s *F*(2, 165.16) = 6.37, *p* < 0.01, ω^2^≈ 0.055. The meritocratic group (*M* = 3.29, *SD* = 1.01) scored higher than both the non-meritocratic (*M* = 2.81, *SD* = 0.75, *p* < 0.01) and the control group (*M* = 2.94, *SD* = 0.78, *p* < 0.05), with no difference between the latter two.

For self-stigma, the ANOVA was significant, *F*(2, 251) = 40.98, *p* < 0.001, η^2^ = 0.246. The meritocratic group (*M* = 4.03, *SD* = 0.84) scored higher than the control (*M* = 3.35, *SD* = 0.82, *p* < 0.001) and non-meritocratic groups (*M* = 2.92, *SD* = 0.74, *p* < 0.001); the control group also scored higher than the non-meritocratic group (*p* < 0.01).

#### Mediation analysis

To test the hypothesized chain mediation model with a multicategorical independent variable, we followed the approach recommended by Fang et al. (2017), using the control group as the reference. Two dummy variables were created: X_1_(meritocratic = 1, control = 0) and X_2_ (non-meritocratic = 1, control = 0). Gender, offense type, and other relevant demographic variables were included as covariates. All variance inflation factors were below 3.01, indicating no serious multicollinearity. FDR correction (*q* < 0.05) was applied to all pairwise comparisons. The analysis was conducted in the R package lavaan, with 5,000 bias-corrected bootstrap resamples; a 95% confidence interval excluding zero indicated a significant effect.

Meritocratic climate (vs. control). The regression paths are shown in Figure 3. The meritocratic climate dummy positively predicted social disconnection (β = 0.212, *p* < 0.01) and self-stigma (β = 0.292, *p* < 0.001), and social disconnection positively predicted self-stigma (β = 0.357, *p* < 0.001). However, the meritocratic climate dummy did not significantly predict collective self-esteem, nor did collective self-esteem significantly predict social disconnection.

**FIGURE 3:**
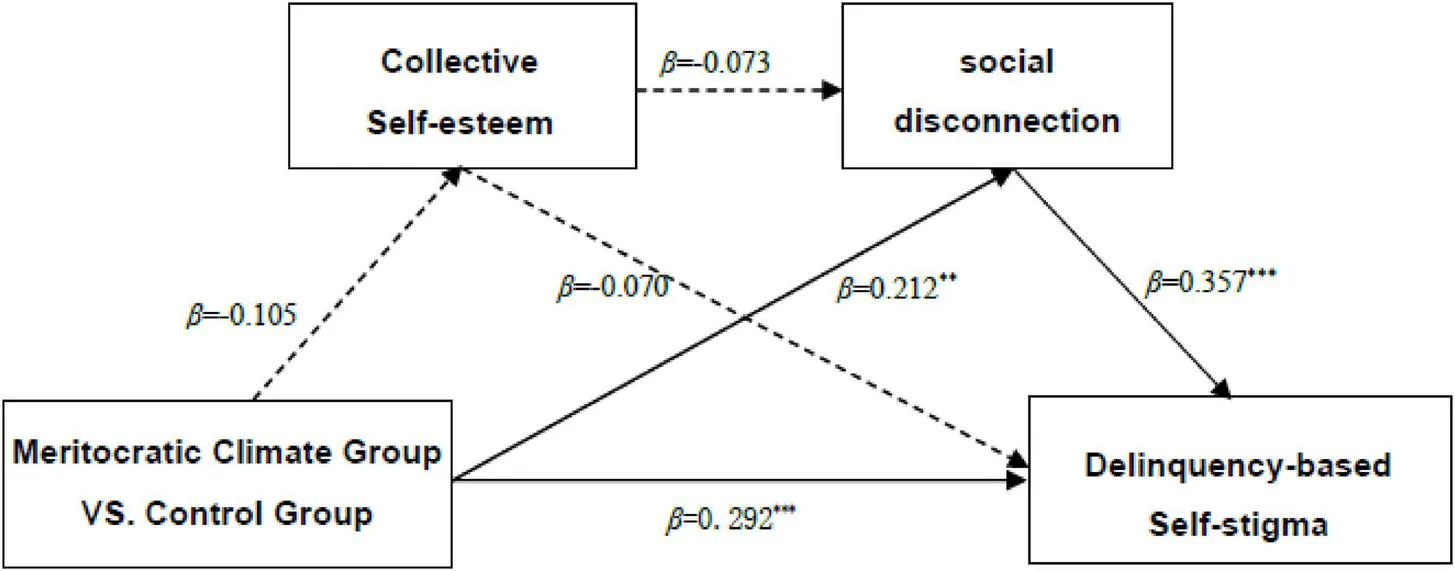
Mediation model of social disconnection in the effects of primed social meritocratic climate on self-stigma among justice-involved adolescents. **p < 0.01, ***p < 0.001.

Table 7 presents the decomposition of effects. The total effect of meritocratic climate (vs. control) on self-stigma was significant (*b* = 0.723, 95%CI [0.459, 0.986]), as was the direct effect (*b* = 0.559, 95%CI [0.309, 0.802]), accounting for 77.32% of the total effect. The total indirect effect was significant (*b* = 0.164, 95%CI [0.056, 0.292], 22.68% of total). Of the specific indirect paths, only the path via social disconnection was significant (*b* = 0.145, 95% CI [0.042, 0.267]), explaining 70.47% of the total indirect effect. Neither the path through collective self-esteem alone nor the serial path (collective self-esteem → social disconnection) was significant. Thus, in the experimentally induced meritocratic climate, social disconnection functioned as the sole significant mediator of the heightened self-stigma.

**TABLE 7:** Mediation effects for the meritocratic climate condition (vs. control).

Effect type and path	*b*	Proportion	95% CI
Total effect decomposition		(% of total effect)	
Total effect	0.723	100%	[0.459, 0.986]
Direct effect	0.559	77.32%	[0.309, 0.802]
Total indirect effect	0.164	22.68%	[0.056, 0.292]
Indirect path decomposition		(% of indirect effect)	
Path 1	0.014	9.07%	[–0.009, 0.062]
Path 2	0.145	70.47%	[0.042, 0.267]
Path 3	0.005	19.46%	[–0.005, 0.025]

Path 1 = Perceived meritocratic climate → Collective self-esteem → Self-stigma; Path 2 = Perceived meritocratic climate → Social disconnection → Self-stigma; Path 3 = Perceived meritocratic climate → Collective self-esteem → Social disconnection → Self-stigma.

Non-meritocratic climate (vs. control). The non-meritocratic climate dummy exerted a significant total effect on self-stigma (β = -0.365, 95% CI [-0.621, -0.114]), largely due to its direct effect (β = -0.335, 95% CI [-0.583, -0.090]). The total indirect effect was not significant, indicating that the protective effect of the non-meritocratic climate on self-stigma did not operate through the collective self-esteem and social disconnection pathways examined here; rather, it may reflect a more direct reduction in self-stigma or involve other unmeasured mechanisms.

Collectively, these findings demonstrate that a meritocratic climate intensifies self-stigma primarily by increasing social disconnection, whereas a non-meritocratic climate reduces self-stigma directly, without engaging the proposed mediation chain.

### Discussion

Study 2 experimentally tested the effects of primed meritocratic versus non-meritocratic social climates on self-stigma among justice-involved adolescents, along with the mediating roles of collective self-esteem and social disconnection. The two climates produced opposite effects: a meritocratic climate increased self-stigma, whereas a non-meritocratic climate reduced it, supporting Hypothesis 1. The meritocratic climate’s negative effect was mediated solely by heightened social disconnection; collective self-esteem played no significant mediating role, either independently or in the chain, thus only partially supporting Hypothesis 2. The non-meritocratic climate exerted a direct protective effect without relying on these mediators.

In the meritocratic climate, the emphasis on personal merit likely triggered social identity threat, rapidly evoking social disconnection and thereby intensifying self-stigma. The non-significant mediating role of collective self-esteem may reflect two dynamics. First, under acute threat, collective self-esteem may function defensively—marked by ambivalence between acknowledging group disadvantage and resisting its internalization—making this cognitive pathway less immediate than emotional disconnection (Issmer et al., 2013). Second, the two mediating routes may compete, with the threat to social connectedness overshadowing any buffering effect of collective self-esteem; thus, emotional disconnection is more readily activated and more proximal to self-stigma under acute pressure. This does not negate the potential protective role of collective self-esteem in chronic, naturalistic contexts, as suggested by Study 1, but indicates that its effect may be limited under short-term, experimentally induced threat.

The beneficial effect of the non-meritocratic climate suggests that an inclusive social environment may directly mitigate self-stigma by conveying de-stigmatizing cues and fostering psychological safety (Marinucci et al., 2022). When structural barriers are perceived as less rigid, individuals’ sense of control and certainty may increase, bolstering confidence in overcoming adversity (Tetzlaff and Rothe-Wulf, 2025). Notably, this direct reduction contrasts with the mediated pathway observed for the meritocratic climate, highlighting the distinct psychological processes activated by different climate frames.

Taken together, these experimental findings demonstrate that, relative to the non-meritocratic climate frame, the meritocratic climate frame intensified self-stigma primarily by heightening social disconnection, whereas the non-meritocratic climate frame directly reduced it. Given that the two primes differed on multiple dimensions beyond meritocracy, these findings should be interpreted as reflecting the effects of contrasting societal logics rather than the unique effect of meritocracy *per se*.

## General discussion

Study 1 revealed a significant exploratory chain mediation: perceived meritocratic climate → lower collective self-esteem → greater social disconnection → increased self-stigma. In Study 2, however, only social disconnection mediated the effect under the acute meritocratic prime, and the chain path was not significant. One possible account is that under strong threat, affective isolation is activated earlier than cognitive group evaluation. Nevertheless, the non-replication cautions against treating the serial pathway as an established mechanism.

### Perceived meritocratic climate as a structural risk factor

Both studies consistently showed that a stronger perceived meritocratic climate predicted greater self-stigma. This finding is consistent with critiques that meritocracy, by attributing success solely to personal effort and talent while neglecting structural barriers, can foster self-blame among those who do not meet its standards (Sandel, 2020). For justice-involved adolescents—whose delinquent identity fundamentally conflicts with the socially admired image of a “successful person”—this climate intensifies the perception of being devalued, thereby reinforcing self-stigma. These results support Hypothesis 1.

### Social disconnection as the core mediator

Social disconnection consistently mediated the link between perceived meritocratic climate and self-stigma. In both the cross-sectional and experimental data, a stronger meritocratic climate was associated with heightened feelings of exclusion and estrangement from mainstream groups, which in turn predicted greater self-stigma. This pattern aligns with the social identity threat model (Major and O’Brien, 2005): contextual cues that signal devaluation can trigger social identity threat and subsequent stress responses. It is also consistent with need-to-belong theory (Baumeister and Leary, 1995), which holds that thwarted belongingness increases vulnerability to internalizing negative evaluations. Notably, social disconnection was more directly linked to self-stigma than collective self-esteem, suggesting that the emotional experience of disconnection may be a more proximal predictor of self-stigma in this population. Supplementary analyses treating the two dimensions separately yielded a consistent pattern: social disconnection mediated the link between each dimension and self-stigma, supporting the centrality of social disconnection as a mediator.

### The conditional and indirect role of collective self-esteem

Neither study found an independent mediating effect of collective self-esteem. Instead, its influence was transmitted through social disconnection. Study 1 revealed a significant chain mediation: perceived meritocratic climate → lower collective self-esteem → greater social disconnection → increased self-stigma. In Study 2, however, only social disconnection mediated the effect under the acute meritocratic prime, and the chain path was not significant. Under strong threat, affective isolation is activated earlier than cognitive group evaluation and more directly drives self-stigma exacerbation (Tetzlaff and Rothe-Wulf, 2025; Marinucci et al., 2022). One possible interpretation for this divergence is that the cross-sectional design of Study 1 captured cumulative, long-term effects of living under a pervasive meritocratic climate, whereas the brief experimental manipulation in Study 2 primarily activated immediate emotional responses such as social disconnection. However, because the serial pathway was exploratory and was not replicated experimentally, this explanation remains tentative and should be tested in future research.

Taken together, these findings highlight that a meritocratic climate intensifies self-stigma primarily by eroding social connectedness. Regardless of whether collective self-esteem is engaged, the emotional experience of disconnection from mainstream society appears to be the critical pathway. Consequently, interventions for justice-involved adolescents might prioritize restoring social bonds—for example, through caring relationships and inclusive group memberships—alongside longer-term efforts to cultivate positive collective identities. By demonstrating both correlational and causal evidence, these two studies clarify the psychological mechanisms through which a widely endorsed meritocratic ideology can harm those it marginalizes.

## Limitations

Drawing on Sandel’s (2020) critique, we introduce the concept of perceived societal meritocratic climate—individuals’ subjective sense that society sorts people into “winners” and “losers” and credits personal merit for their outcomes. Our finding that this perception heightens self-stigma among justice-involved adolescents aligns with evidence that salient meritocratic norms can foster internal attributions and negative stereotypes of low-status groups (Madeira et al., 2019). Importantly, whereas prior work has typically manipulated meritocratic beliefs from the public’s perspective, the present research foregrounds how low-status individuals themselves perceive and interpret the meritocratic logic surrounding them.

Several limitations of the present research must be acknowledged. First, the conceptual fuzziness of meritocracy remains a challenge. Despite our efforts to differentiate perceived societal meritocratic climate from related constructs, meritocracy is inherently multifaceted and even contradictory: it overlaps with belief in a just world, system justification, work ethic, and social mobility beliefs, such that a person can simultaneously endorse “hard work leads to success” and doubt that upward mobility is attainable. This complexity complicates both scale development and the design of valid priming materials.

Second, our operationalization of meritocratic versus non-meritocratic climates warrants caution. The primes did not activate personal meritocratic beliefs *per se*; rather, they depicted a societal reality governed by meritocratic logic—glorifying winners and denigrating losers—or a contrasting climate that recognizes structural constraints and diverse values. The non-meritocratic condition thus represents an ideal-type vision in which, as Sandel (2020) argues, true meritocracy is accepted as unattainable and individual blame is seen as undermining social solidarity. Relatedly, it is important to acknowledge that the two experimental primes differed on multiple dimensions beyond meritocracy. The meritocratic prime emphasized achievement, personal responsibility, and derogation of “losers,” whereas the non-meritocratic prime emphasized diversity, personal meaning, happiness, social good, and inclusion. Thus, social inclusion, attributional framing, and anticipated exclusion were varied simultaneously with meritocracy. Consequently, the observed effects cannot be uniquely attributed to meritocracy per se; rather, they should be interpreted as reflecting the consequences of contrasting climate frames. Future experimental work should use more granular manipulations that isolate attribution, social inclusion, and anticipated exclusion from meritocratic content to test their independent contributions.

Third, the generalizability of our findings is constrained not only by sample homogeneity but also by unexamined cultural dynamics. Our participants were drawn primarily from specialized schools in China, with limited diversity in geographic region, offense type, and socioeconomic background. More importantly, this sampling context is embedded in a cultural mainstream narrative that “hard work ensures success regardless of origin or talent.” Such a narrative likely fuels public stereotypes of justice-involved adolescents as lazy, incompetent, or morally deficient, while obscuring the structural constraints they face—insufficient family resources, lack of parental care, and weak institutional support. When outcomes are evaluated in isolation from these constraints, the result is an “outcome-based” attribution of responsibility (Andre, 2025) that may deepen self-stigma through public exclusion and devaluation. Our design did not capture how this macro-level cultural discourse penetrates adolescents’ subjective perceptions of meritocracy, nor how it interacts with individual-level beliefs. Future research should therefore recruit justice-involved youth from diverse judicial settings and employ cross-cultural designs to disentangle the pathways through which broad societal narratives shape personal perceptions of meritocracy and self-stigma.

Fourth, the serial mediation model involving collective self-esteem and social disconnection was developed *post-hoc* during the revision process and was not pre-registered. Although the chain pathway was significant in Study 1, it was not replicated in Study 2. Therefore, this pathway should be regarded as exploratory and requires confirmatory evidence from pre-registered, longitudinal, or experimental designs before strong mechanistic conclusions can be drawn.

Beyond these specific concerns, future studies should examine how perceived meritocratic climate interacts with individual value-belief systems to shape downstream behaviors and attitudes. Although our cross-sectional and experimental evidence establishes a robust link between perceived societal meritocratic climate and self-stigma, the key mediating mechanisms remain to be clarified. In sum, alleviating self-stigma among justice-involved adolescents may ultimately require a shift in societal emphasis from narrow “meritocratic success” to genuine respect for human worth—even for those who have transgressed.

## Data Availability

The raw data supporting the conclusions of this article will be made available by the authors, without undue reservation.

## References

[B1] Andre P. (2025). Shallow meritocracy. *Rev. Econ. Stud.* 92, 772–807. 10.1093/restud/rdae040

[B2] Bai X. F. You H. J. (2025). *Building an Integrated System of “punishment, Correction, and Education” to Prevent Juvenile Delinquency. Procuratorial Daily.* Available online at; https://www.spp.gov.cn/spp/llyj/202503/t20250327_691576.shtml (accessed March 27, 2025)

[B3] Baumeister R. F. Leary M. R. (1995). The need to belong: Desire for interpersonal attachments as a fundamental human motivation. *Psychol. Bull.* 117, 497–529. 10.1037/0033-2909.117.3.497 7777651

[B4] Baumeister R. F. Brewer L. E. Tice D. M. Twenge J. M. (2007). Thwarting the need to belong: Understanding the interpersonal and inner effects of social exclusion. *Soc. Pers. Psychol. Compass* 1, 506–520. 10.1111/j.1751-9004.2007.00020.x

[B5] Bohren J. A. Hull P. Imas A. (2025). Systemic discrimination: Theory and measurement. *Quart. J. Econ.* 140, 1743–1799. 10.1093/qje/qjaf022

[B6] Dalbert C. (1999). The world is more just for me than generally: About the personal belief in a just world scale’s validity. *Soc. Justice Res.* 12, 79–98. 10.1023/A:1022091609047

[B7] Darnon C. Smeding A. Redersdorff S. (2018). Belief in school meritocracy as an ideological barrier to the promotion of equality. *Eur. J. Soc. Psychol.* 48, 523–534. 10.1002/ejsp.2347

[B8] Day M. V. Fiske S. T. (2017). Movin’ on up? How perceptions of social mobility affect our willingness to defend the system. *Soc. Psychol. Pers. Sci.* 8, 267–274. 10.1177/1948550616678454 30766651 PMC6371776

[B9] Di X. H. (2012). The theoretical basis of juvenile justice: The particularity of juveniles. *J. Shandong Police Coll.* 24, 49–54.

[B10] Ellemers N. Spears R. Doosje B. (2002). Self and social identity. *Annu. Rev. Psychol.* 53, 161–186. 10.1146/annurev.psych.53.100901.135228 11752483

[B11] Fan X. L. Wei J. Zhang J. F. (2015). Reliability and validity of the revised social connectedness scale in middle school students. *J. Southwest China Normal Univer.* 40, 118–122. 10.13718/j.cnki.xsxb.2015.08.022

[B12] Fang J. Wen Z. L. Zhang M. Q. (2017). Mediation effect analysis of categorical variables. *Psychol. Sci.* 40, 471–477. 10.16719/j.cnki.1671-6981.20170233

[B13] Faul F. Erdfelder E. Buchner A. Lang A.-G. (2009). Statistical power analyses using G*Power 3.1: Tests for correlation and regression analyses. *Behav. Res. Methods* 41, 1149–1160. 10.3758/BRM.41.4.1149 19897823

[B14] García-Sierra A. (2023). The dark side of meritocratic beliefs: Is believing in meritocracy detrimental to individuals from low socioeconomic backgrounds? *Soc. Just. Res.* 36, 385–409. 10.1007/s11211-023-00413-x

[B15] Gilbert K. M. (2023). *How Do Meritocracy Beliefs Shape Motivation? An Exploration of Meritocracy Beliefs, Perceived Competitiveness, and Achievement Goals.* Doctoral dissertation. New York, NY: University of Rochester.

[B16] Hayes A. F. (2013). *Introduction to Mediation, Moderation, and Conditional Process Analysis: A Regression-Based Approach.* New York, NY: Guilford Press.

[B17] Hogg M. A. Abrams D. (2011). *Social Identity Processes (Chinese Translation).* Beijing: China Renmin University Press.

[B18] Hoyt C. L. Burnette J. L. Forsyth R. B. Parry M. DeShields B. H. (2021). Believing in the American dream sustains negative attitudes toward those in poverty. *Soc. Psychol. Quart.* 84, 203–215. 10.1177/01902725211022319

[B19] Huang C. (2019). *Research on the Reintegration of Released Juvenile Offenders Into Society: An Analytical Perspective of Social Integration.* Doctoral dissertation. Wuhan: Central China Normal University.

[B20] Issmer C. Stellmacher J. Gollwitzer M. (2013). When disadvantaged adolescents strike out: The impact of negative metastereotypes on delinquency. *J. Crim. Psychol.* 3, 4–18. 10.1108/20093821311307721

[B21] Jetten J. Branscombe N. R. Haslam S. A. Haslam C. Cruwys T. Jones J. M. et al. (2015). Having a lot of a good thing: Multiple important group memberships as a source of self-esteem. *PLoS One* 10:e0124609. 10.1371/journal.pone.0124609 26017554 PMC4446320

[B22] Koo H. J. Piff P. K. Shariff A. F. (2023). If I could do it, so can they: Among the rich, those with humbler origins are less sensitive to the difficulties of the poor. *Soc. Psychol. Pers. Sci.* 14, 333–341. 10.1177/19485506221098921 36844784 PMC9947719

[B23] Koo H. J. Piff P. K. Moskowitz J. P. Shariff A. F. (2024). System circumvention: Dishonest-illegal transgressions are perceived as justified in non-meritocratic societies. *Br. J. Soc. Psychol.* 63, 1565–1586. 10.1111/bjso.12737 38456665

[B24] Kuppens T. Spears R. Manstead A. S. R. Spruyt B. Easterbrook M. J. (2018). Educationism and the irony of meritocracy: Negative attitudes of higher educated people towards the less educated. *J. Exp. Soc. Psychol.* 76, 429–447. 10.1016/j.jesp.2017.11.001

[B25] Laurin K. Fitzsimons G. M. Kay A. C. (2011). Social disadvantage and the self-regulatory function of justice beliefs. *J. Pers. Soc. Psychol.* 100, 149–171. 10.1037/a0021343 21058869

[B26] Lee R. M. Draper M. Lee S. (2001). Social connectedness, dysfunctional interpersonal behaviors, and psychological distress: Testing a mediator model. *J. Counseling Psychol.* 48, 310–318. 10.1037/0022-0167.48.3.310

[B27] Littler J. (2018). *Against Meritocracy: Culture, Power and Myths of Mobility.* Milton Park: Routledge.

[B28] Liu Y. (2024). The dual legal attributes and reform path of specialized education. *J. Youth Stud.* 43, 129–140. 10.16034/j.cnki.10-1318/c.2024.05.012

[B29] Longares L. Escartín J. Rodríguez-Carballeira Á (2016). Collective self-esteem and depressive symptomatology in lesbians and gay men: A moderated mediation model of self-stigma and psychological abuse. *J. Homosexuality* 63, 1481–1501. 10.1080/00918369.2016.1223333 27533337

[B30] Luhtanen R. Crocker J. (1992). A collective self-esteem scale: Self-evaluation of one’s social identity. *Pers. Soc. Psychol. Bull.* 18, 302–318. 10.1177/0146167292183006

[B31] Lyu X. Zhong N. Tian B. et al. (2017). The effect of different types of intergroup threat on collective self-esteem: The mediating role of anger and inferiority. *Psychol. Sci.* 40, 632–637. 10.16719/j.cnki.1671-6981.20170319

[B32] Madeira A. F. Costa-Lopes R. Dovidio J. F. Freitas G. Mascarenhas M. F. (2019). Primes and consequences: A systematic review of meritocracy in intergroup relations. *Front. Psychol.* 10:2007. 10.3389/fpsyg.2019.02007 31607971 PMC6761281

[B33] Major B. O’Brien L. T. (2005). The social psychology of stigma. *Annu. Rev. Psychol.* 56, 393–421. 10.1146/annurev.psych.56.091103.070137 15709941

[B34] Marinucci M. Mazzoni D. Pancani L. Riva P. (2022). To whom should I turn? Intergroup social connections moderate social exclusion’s short- and long-term psychological impact on immigrants. *J. Exp. Soc. Psychol.* 99:104275. 10.1016/j.jesp.2021.104275

[B35] McCoy S. K. Wellman J. D. Cosley B. Saslow L. Epel E. (2013). Is the belief in meritocracy palliative for members of low status groups? Evidence for a benefit for self-esteem and physical health via perceived control. *Eur. J. Soc. Psychol.* 43, 307–318. 10.1002/ejsp.1959 24039310 PMC3769703

[B36] Morris K. Bühlmann F. Sommet N. Vandecasteele L. (2022). The paradox of local inequality: Meritocratic beliefs in unequal localities. *Br. J. Sociol.* 73, 421–460. 10.1111/1468-4446.12930 35261026 PMC9314027

[B37] Pan Z. D. Li A. R. (2026). The worry of meritocracy: School competition environment and adolescent bullying from a global perspective. *Youth Stud.* 11, 223–224. 10.27017/j.cnki.qnyj.2026.01.011

[B38] Quinn D. M. Crocker J. (1999). When ideology hurts: Effects of belief in the Protestant ethic and feeling overweight on the psychological well-being of women. *J. Pers. Soc. Psychol.* 77, 402–414. 10.1037/0022-3514.77.2.402 10474214

[B39] Rauscher C. Louvet E. Bourguignon D. (2024). “To succeed at work, you just need to work hard”: How beliefs in professional meritocracy serve to blame unemployed people. *J. Commun. Appl. Soc. Psychol.* 34, e2762. 10.1002/casp.2762

[B40] Rüsch N. Todd A. R. Bodenhausen G. V. Corrigan P. W. (2010). Biogenetic models of psychopathology, implicit guilt, and mental illness stigma. *Eur. Arch. Psychiatry Clin. Neurosci.* 260, 617–625. 10.1007/s00406-010-0111-4 20493559 PMC2928405

[B41] Sandel M. J. (2020). *The Tyranny of Merit: What’s Become of the Common Good?*, 1st Edn. New York, NY: Farrar, Straus and Giroux.

[B42] Son Hing L. S. Bobocel D. R. Zanna M. P. (2002). Meritocracy and opposition to affirmative action: Making concessions in the face of discrimination. *J. Pers. Soc. Psychol.* 83, 493–509. 10.1037/0022-3514.83.3.493 12219850

[B43] Son Hing L. S. Bobocel D. R. Zanna M. P. Garcia D. M. Gee S. S. Orazietti K. (2011). The merit of meritocracy. *J. Pers. Soc. Psychol.* 101, 433–450. 10.1037/a0024618 21787093

[B44] Tetzlaff J. Rothe-Wulf A. (2025). Barriers to belonging: Influences of social exclusion beyond group relations. *Curr. Psychol.* 44, 17751–17758. 10.1007/s12144-025-08409-3

[B45] Tomova Shakur T. K. Phillips L. T. (2022). What counts as discrimination? How principles of merit shape fairness of demographic decisions. *J. Pers. Soc. Psychol. Interpersonal Relat. Group Process.* 123, 957–982. 10.1037/pspi0000383 35025597

[B46] Trevisan F. Rusconi P. Hanna P. Hegarty P. (2022). Psychologising meritocracy: A historical account of its many guises. *Theory Psychol.* 32, 221–242. 10.1177/09593543211057098

[B47] Wu C. Z. Wang T. Yu L. X. (2020). The relationship between depression and suicide attempt: The mediating role of psychological pain and the moderating role of social connectedness. *Chinese J. Clin. Psychol.* 28, 679–682. 10.16128/j.cnki.1005-3611.2020.04.006

[B48] Wu S. W. Gao D. S. (2023). Reflection on educational meritocracy: Significance of progress, moral risks, and mitigation strategies. *Glob. Educ.* 52, 36–49.

[B49] Yao J. L. Liu X. (2024). Maintaining rational expectations for specialized education. *J. Youth Stud.* 43, 106–117. 10.16034/j.cnki.10-1318/c.2024.05.013

[B50] Yao J. L. Shen C. Z. (2025). Legal attributes and necessary characteristics of special correctional education. *Legal Forum* 40, 90–101.

[B51] Young M. (1994). *The Rise of the Meritocracy.* Milton Park: Routledge, 10.4324/9781315134642

[B52] Zhang L. W. Liang Z. P. (2002). Athletes’ life satisfaction: The contribution of individual self-esteem and collective self-esteem. *Acta Psychol. Sin.* 34, 160–167.

[B53] Zhou H. Long L. R. (2004). Statistical tests and control methods for common method variance. *Adv. Psychol. Sci.* 6, 942–950. 10.3969/j.issn.1671-3710.2004.06.018

