# Supplementary material for: Perceived societal meritocratic climate and self-stigma among adolescents in conflict with the law: the mediating role of collective self-esteem and social disconnection
Source: Front Psychol. 2026 Sep 15;17:1852343. doi: 10.3389/fpsyg.2026.1852343 (PMC13619910; doi:10.3389/fpsyg.2026.1852343)
Supplement: Supplementary file 1 [file Supplementary_file_1.docx]

# SUPPLEMENTARY MATERIALS

**PART 1：Perceived Societal Meritocratic Climate Scale (Independent Variable)**

**1.1 Rationale for Scale Development**

We appreciate the reviewer's concern about using a newly developed measure. The development of this scale was motivated by the following considerations: (1) existing stigma scales are predominantly adapted from the mental illness literature and primarily validated with adult offenders; (2) juvenile offenders represent a distinct population with unique developmental characteristics (adolescence) and specific stigmatization experiences related to their offending identity; (3) no validated instrument specifically captures the multidimensional nature of self-stigma among Chinese juvenile offenders. Therefore, we followed rigorous psychometric procedures to develop and validate this scale. A separate manuscript detailing the full scale development process is currently under review.

**1.2 Item Analysis**

Table 1 Item Analysis Results（*n*=440）

| Item | CR | Corrected Item-Total Correlation | Cronbach's Alpha if Item Deleted | Communality | Factor Loading | Note |
| --- | --- | --- | --- | --- | --- | --- |
| 1 | 12.507^***^ | 0.578^**^ | 0.912 | 0.371 | 0.609 | Retained |
| 2 | 18.559^***^ | 0.713^**^ | 0.909 | 0.551 | 0.743 | Retained |
| 3 | 8.687^***^ | 0.509^**^ | 0.914 | 0.250 | 0.500 | Retained |
| 4 | 12.058^***^ | 0.650^**^ | 0.911 | 0.388 | 0.623 | Retained |
| 5 | 13.369^***^ | 0.674^**^ | 0.910 | 0.495 | 0.704 | Retained |
| 6 | 15.651^***^ | 0.682^**^ | 0.910 | 0.498 | 0.706 | Retained |
| 7 | **1.815** | **0.211^**^** | **0.920** | **0.011** | **0.104** | Deleted |
| 8 | 10.567^***^ | 0.594^**^ | 0.912 | 0.294 | 0.542 | Retained |
| 9 | 16.079^***^ | 0.666^**^ | 0.910 | 0.463 | 0.680 | Retained |
| 10 | 4.923^***^ | **0.365^**^** | **0.917** | **0.071** | **0.267** | Deleted |
| 11 | 18.408^***^ | 0.763^**^ | 0.908 | 0.642 | 0.801 | Retained |
| 12 | 10.001^***^ | 0.624^**^ | 0.911 | 0.363 | 0.602 | Retained |
| 13 | 18.446^***^ | 0.726^**^ | 0.909 | 0.578 | 0.761 | Retained |
| 14 | 12.388^***^ | 0.624^**^ | 0.911 | 0.360 | 0.600 | Retained |
| 15 | 9.581^***^ | 0.579^**^ | 0.912 | 0.276 | 0.525 | Retained |
| 16 | 4.888^***^ | **0.387^**^** | **0.917** | **0.092** | **0.304** | Deleted |
| 17 | 16.592^***^ | 0.672^**^ | 0.910 | 0.484 | 0.696 | Retained |
| 18 | 15.601^***^ | 0.704^**^ | 0.910 | 0.546 | 0.739 | Retained |
| 19 | 14.893^***^ | 0.702^**^ | 0.910 | 0.523 | 0.723 | Retained |
| 20 | 9.396^***^ | 0.541^**^ | 0.913 | 0.279 | 0.528 | Retained |
| 21 | 12.753^***^ | 0.656^**^ | 0.911 | 0.489 | 0.699 | Retained |
| 22 | 11.812^***^ | 0.605^**^ | 0.912 | 0.416 | 0.645 | Retained |

*Notes*: 1. **p* < 0.05, ***p* < 0.01, ****p* < 0.001; 2. The criteria for item analysis were set as follows: Critical Ratio (CR) ≥ 3.000, Corrected Item-Total Correlation ≥ 0.400, Cronbach's Alpha if Item Deleted ≤ overall scale reliability (0.916), Communality ≥ 0.20, and Factor Loading ≥ 0.45; 3. Based on these criteria, Items 7, 10, and 16 were deleted, while the remaining 19 items were retained for subsequent exploratory factor analysis.

**1.3 Exploratory Factor Analysis**

The remaining 19 items were subjected to exploratory factor analysis (EFA). The KMO measure was 0.933, and Bartlett's test of sphericity was significant (*χ*² = 4289.147, *df* = 171, *p* < 0.001), confirming the factorability of the data. Factors were extracted using maximum likelihood estimation with Promax oblique rotation.

Table 2 Exploratory Factor Analysis (*n*=440)

| Item | Rotated Factor Loadings | | Communality |
| --- | --- | --- | --- |
|  | Factor1 | Factor2 |  |
| 11 In society, losers are easily looked down upon or belittled. | 0.83 |  | 0.69 |
| 2 In society, people without educational qualifications or diplomas are more likely to suffer from discrimination or unfair treatment | 0.79 |  | 0.59 |
| 5 In school, high-achieving students are recognized and included, and are more likely to receive preferential treatment and opportunities. | 0.73 |  | 0.50 |
| 13 In contemporary society, people look down on those with low levels of education. | 0.73 |  | 0.60 |
| 21 Those from affluent family backgrounds are more likely to obtain resources and opportunities for success. | 0.71 |  | 0.49 |
| 22 In society, successful people are more likely to gain respect, status, and reputation. | 0.71 |  | 0.42 |
| 18 In society, poverty or lack of education is likely to be looked down upon. | 0.71 |  | 0.53 |
| 6 In society, most people look down on those who lack ability and are not diligent enough. | 0.66 |  | 0.48 |
| 9 In school, low-achieving students are considered unintelligent or not hardworking enough, and are thus belittled or ridiculed. | 0.64 |  | 0.47 |
| 17 Both in society and in the family, failures have low social status or little say. | 0.63 |  | 0.47 |
| 15 Most failures in life are due to one's own lack of effort or laziness. |  | 0.82 | 0.56 |
| 8 In contemporary society, failures are generally perceived as lacking in ability or not putting in enough effort. |  | 0.79 | 0.54 |
| 4 Those who fail to pursue higher education or attend college are primarily considered to be not working hard enough or not intelligent enough. |  | 0.63 | 0.50 |
| 12 The efforts or personal abilities of failures are likely to be inferior to those of successful people. |  | 0.58 | 0.45 |
| 14 In contemporary society, successful people achieve success mainly because of their strong personal abilities or sufficient effort. |  | 0.49 | 0.38 |
| Eigenvalue | 6.48 | 1.20 |  |
| Cumulative Variance Explained (%) | 43.18 | 51.18 |  |

**1.3 Confirmatory Factor Analysis (CFA)**

To further validate the factor structure, we conducted a confirmatory factor analysis (CFA) on an independent sample (Sample 2, *n* = 361) using the revised formal scale derived from the exploratory factor analysis.

Table 3 Confirmatory Factor Analysis Fit Indices Scale (*n*=361)

| Fit Index | *χ*2 | *df* | *χ*2/*df* | *RMSEA* | *SRMR* | *CFI* | *TLI* |
| --- | --- | --- | --- | --- | --- | --- | --- |
| Value | 176.79 | 78 | 2.27 | 0.059 | 0.045 | 0.957 | 0.942 |

*Note*: *χ*²/*df* < 3 indicates good fit, < 5 acceptable; *RMSEA* < 0.05 indicates good fit, < 0.08 acceptable; *SRMR* < 0.05 indicates good fit, < 0.08 acceptable; *CFI* and *TLI* > 0.95 indicate good fit, > 0.90 acceptable

To further examine the applicability of the scale among delinquent adolescents, a confirmatory factor analysis (CFA) was also conducted using an independent sample of delinquent adolescents (Sample 3, n = 260). The model fit indices were as follows: χ²/df = 1.88, RMSEA = 0.058, CFI = 0.934, TLI = 0.911, and SRMR = 0.053, indicating acceptable to good model fit across all indices.

**1.4 Convergent Validity (AVE & CR)**

The CR values for the two factors (0.899 and 0.788) were well above the 0.70 benchmark, indicating adequate internal consistency. By contrast, the AVE values (0.473 and 0.434) were slightly below the 0.50 ideal, suggesting that some measurement error remains. That said, as both AVE estimates exceeded the 0.40 threshold deemed acceptable in the context of satisfactory CRs (Fornell & Larcker, 1981), and given the exploratory nature of this scale development study, we interpret these findings as providing preliminary, albeit not conclusive, support for convergent validity. Future research with refined item pools is warranted to further strengthen the scale's psychometric properties.

Table 4 Convergent Validity Indices

| Factor | Composite Reliability (CR) | Average Variance Extracted (AVE) |
| --- | --- | --- |
| Perceived Meritocratic Evaluation Climate (M1) | 0.899 | 0.473 |
| Perceived Meritocratic Attribution Tendency (M2) | 0.788 | 0.434 |

**1.5 Discriminant Validity**

The correlation coefficient between the two factors was 0.565, whereas the square roots of the AVE for M1 and M2 were 0.687 and 0.659, respectively—both exceeding the inter-factor correlation. This satisfies the Fornell-Larcker criterion, indicating that the Perceived Meritocratic Evaluation Climate and Perceived Meritocratic Attribution Tendency, while interrelated, are empirically distinguishable constructs. Furthermore, the heterotrait-monotrait (HTMT) ratio was 0.580, well below the conservative threshold of 0.85 recommended by Henseler et al. (2015), further supporting the robustness of the scale's discriminant validity.

Table 5 Fornell-Larcker Criterion (√AVE vs. Correlations)

| Factor | 1 | 2 | √AVE |
| --- | --- | --- | --- |
| M1 |  |  | 0.687 |
| M2 | 0.565 |  | 0.659 |

Table 6 HTMT Ratio

| Factor | 1 | 2 |
| --- | --- | --- |
| M1 |  |  |
| M2 | 0.580 |  |

**PART 2：Delinquency-based Self-stigma Scale (Dependent Variable)**

**2.1Item Analysis**

Table 6 Summary of Item Analysis

| Item | CR | Corrected Item-Total Correlation | Cronbach's Alpha if Item Deleted | Communality | Factor Loading | Remarks |
| --- | --- | --- | --- | --- | --- | --- |
| 1 | 9.768*** | 0.390** | 0.880 | 0.200 | 0.450 | Retained |
| 2 | 6.483*** | 0.271** | 0.881 | 0.031 | 0.175 | Deleted |
| 3 | -0.677 | -0.026 | 0.886 | 0.021 | -0.144 | Deleted |
| 4 | 5.513*** | 0.239** | 0.882 | 0.010 | 0.098 | Deleted |
| 5 | 10.753*** | 0.410** | 0.879 | 0.215 | 0.464 | Retained |
| 6 | 15.703*** | 0.548** | 0.877 | 0.401 | 0.634 | Retained |
| 7 | 15.193*** | 0.538** | 0.877 | 0.397 | 0.630 | Retained |
| 8 | 15.449*** | 0.533** | 0.877 | 0.395 | 0.629 | Retained |
| 9 | -3.717*** | -0.170** | 0.888 | 0.025 | -0.157 | Deleted |
| 10 | 17.185*** | 0.558** | 0.877 | 0.375 | 0.612 | Retained |
| 11 | 13.199*** | 0.504** | 0.878 | 0.347 | 0.589 | Retained |
| 12 | 9.905*** | 0.419** | 0.879 | 0.243 | 0.493 | Retained |
| 13 | 16.046*** | 0.540** | 0.877 | 0.345 | 0.588 | Retained |
| 14 | 10.104*** | 0.389** | 0.880 | 0.062 | 0.249 | Deleted |
| 15 | 11.512*** | 0.419** | 0.879 | 0.084 | 0.291 | Deleted |
| 16 | 13.173*** | 0.522** | 0.877 | 0.314 | 0.561 | Retained |
| 17 | 9.246*** | 0.365** | 0.880 | 0.03 | 0.175 | Deleted |
| 18 | 13.189*** | 0.488** | 0.878 | 0.327 | 0.572 | Retained |
| 19 | 7.897*** | 0.304** | 0.881 | 0.081 | 0.285 | Deleted |
| 20 | 8.934*** | 0.358** | 0.880 | 0.094 | 0.307 | Deleted |
| 21 | 14.194*** | 0.499** | 0.878 | 0.289 | 0.538 | Retained |
| 22 | 10.832*** | 0.406** | 0.879 | 0.059 | 0.242 | Deleted |
| 23 | 15.257*** | 0.564** | 0.876 | 0.330 | 0.574 | Retained |
| 24 | 4.729*** | 0.216** | 0.882 | 0.001 | 0.031 | Deleted |
| 25 | 11.807*** | 0.465** | 0.878 | 0.277 | 0.526 | Retained |
| 26 | 11.173*** | 0.468** | 0.878 | 0.216 | 0.465 | Retained |
| 27 | 8.798*** | 0.360** | 0.880 | 0.031 | 0.176 | Deleted |
| 28 | 15.852*** | 0.529** | 0.877 | 0.340 | 0.583 | Retained |
| 29 | 16.050*** | 0.543** | 0.877 | 0.332 | 0.576 | Retained |
| 30 | 15.972*** | 0.561** | 0.877 | 0.407 | 0.638 | Retained |
| 31 | 14.983*** | 0.552** | 0.877 | 0.385 | 0.620 | Retained |
| 32 | 7.914*** | 0.319** | 0.881 | 0.028 | 0.166 | Deleted |
| 33 | 16.336*** | 0.577** | 0.876 | 0.402 | 0.634 | Retained |
| 34 | 12.545*** | 0.464** | 0.878 | 0.276 | 0.526 | Retained |
| 35 | 6.594*** | 0.295** | 0.881 | 0.015 | 0.121 | Deleted |
| 36 | 17.954*** | 0.583** | 0.876 | 0.397 | 0.63 | Retained |
| 37 | 5.719*** | 0.194** | 0.883 | 0.045 | 0.213 | Deleted |
| 38 | 8.120*** | 0.372** | 0.880 | 0.113 | 0.337 | Deleted |
| 39 | 6.722*** | 0.301** | 0.881 | 0.073 | 0.270 | Deleted |
| 40 | 11.625*** | 0.490** | 0.878 | 0.273 | 0.523 | Retained |
| 41 | 6.675*** | 0.293** | 0.881 | 0.016 | 0.128 | Deleted |
| 42 | 8.347*** | 0.350** | 0.880 | 0.033 | 0.182 | Deleted |
| 43 | 8.535*** | 0.326** | 0.881 | 0.090 | 0.300 | Deleted |
| 44 | 10.079*** | 0.390** | 0.879 | 0.042 | 0.206 | Deleted |

Note: ***p*＜0.01，****p*＜0.001.

**2.2 Exploratory Factor Analysis**

Following the item analysis, an exploratory factor analysis (EFA) was conducted on the retained items to simplify the data structure by extracting a smaller number of underlying factors. In this study, the Kaiser–Meyer–Olkin (KMO) measure was 0.927, indicating that the inter‑item correlations were strong and highly suitable for factor analysis. Bartlett's test of sphericity was significant (χ² = 6431.67, df = 253, p < 0.001). The principal component analysis method was employed, and factors with eigenvalues greater than 1.00 were extracted to examine the dimensional structure and item distribution of the scale. This was followed by an oblique rotation using the optimal Promax method. The factor loadings of each item on the different factors and their communalities are presented below.

Table 7 Exploratory Factor Analysis (*n*=720)

| Items | Rotated Factor Loadings | | Communality |
| --- | --- | --- | --- |
|  | Factor1 | Factor 2 |  |
| 11 Most people look down on delinquent adolescents like me. | 0.80 |  | 0.63 |
| 10 Most people think delinquent adolescents like me are untrustworthy and unreliable. | 0.76 |  | 0.57 |
| 7 Most people are unwilling to work with those who were once delinquent adolescents like me. | 0.73 |  | 0.59 |
| 8 Most people feel disgusted with delinquent adolescents like me. | 0.72 |  | 0.60 |
| 12Most people do not value the suggestions or opinions of delinquent adolescents like me. | 0.71 |  | 0.46 |
| 13 Most people think delinquent adolescents like me are incapable of repenting. | 0.69 |  | 0.48 |
| 5 Most people think delinquent adolescents like me need to be severely punished. | 0.59 |  | 0.35 |
| 1 When a delinquent adolescent like me returns to their original environment, people will deliberately keep their distance from him/her. | 0.59 |  | 0.33 |
| 31 I feel inferior due to my offending identity. |  | 0.85 | 0.66 |
| 28 I hate myself because of my offending identity. |  | 0.77 | 0.55 |
| 36 I feel that I will be lonelier due to social exclusion in the future. |  | 0.76 | 0.54 |
| 33 I feel that my offending identity makes me have no social status. |  | 0.70 | 0.49 |
| 30 I feel that I am dangerous to society or to others. |  | 0.69 | 0.54 |
| 29 Even if I sincerely repent, people will not truly trust me. |  | 0.60 | 0.40 |
| 18I worry or fear that others will find out about my offending identity and experiences. |  | 0.59 | 0.42 |
| Eigenvalue | 5.81 | 1.81 |  |
| Cumulative Variance Explained (%) | 38.73 | 50.79 |  |

Factor 1, labeled the Perceived Stigma dimension, comprised 8 items (Items 1, 5, 7, 8, 10, 11, 12, and 13), reflecting delinquent adolescents' perceptions of external discrimination, prejudice, and social alienation directed toward them due to their offending identity. Factor 2, labeled the Internalized Stigma dimension, comprised 7 items (Items 18, 28, 29, 30, 31, 33, and 36), reflecting delinquent adolescents' subjective self‑perceptions, affective experiences, and negative expectations regarding their offending identity, thereby indicating the extent to which they have internalized external labels

**2.3 Confirmatory Factor Analysis (CFA)**

Table 8 Confirmatory Factor Analysis Fit Indices (*n*=356)

| Fit Index | *χ*2 | *df* | *χ*2/*df* | *RMSEA* | *SRMR* | *CFI* | *TLI* |
| --- | --- | --- | --- | --- | --- | --- | --- |
| Value | 175.792 | 86 | 2.044 | 0.054 | 0.044 | 0.959 | 0.950 |

*Note*: χ²/df < 3 indicates good fit, < 5 acceptable; RMSEA < 0.05 good, < 0.08 acceptable; SRMR < 0.05 good, < 0.08 acceptable; CFI and TLI > 0.95 good, > 0.90 acceptable.

The CFA results indicate that all fit indices met or exceeded the recommended criteria, confirming that the four-factor theoretical model fits the observed data well and the scale structure is validated.

**2.4 Convergent Validity (AVE & CR)**

Table 9 Convergent Validity for the Delinquency-based Self-stigma Scale

| Factor | Items | CR | AVE |
| --- | --- | --- | --- |
| Perceived Stigma (PS) | 8 | 0.863 | 0.445 |
| Internalized Stigma (IS) | 7 | 0.854 | 0.456 |

The AVE values for the Perceived Stigma and Internalized Stigma dimensions were 0.445 and 0.458, respectively—marginally below the ideal cut-off of 0.50 but still above the commonly accepted threshold of 0.40. In contrast, the composite reliability (CR) values for the two dimensions were 0.863 and 0.854, respectively, well above the recommended benchmark of 0.70. Taken together, these findings suggest that the convergent validity of the scale is acceptable, though further refinement of the items may be desirable to enhance the AVE estimates in future studies.

**2.5 Discriminant Validity**

Table 10 Fornell-Larcker Criterion (√AVE vs. Correlations)

| Factor | 1 | 2 | √AVE |
| --- | --- | --- | --- |
| 1 PS |  |  | 0.667 |
| 2 IS | 0.650 |  | 0.677 |

Note: Boldfaced diagonal entries are the square roots of the average variance extracted (AVE) for each factor; off‑diagonal entries below the main diagonal are Pearson correlation coefficients among the factors.

The correlation coefficient between the two factors was 0.650, while the square roots of the AVE for the Perceived Stigma and Internalized Stigma dimensions were 0.667 and 0.677, respectively—both exceeding the inter-factor correlation (0.650). This satisfies the Fornell-Larcker criterion (√AVE > inter-factor correlation), indicating adequate discriminant validity between the two factors.

Table 11 HTMT Ratio

| Factor | 1 | 2 |
| --- | --- | --- |
| 1 PS | — |  |
| 2 IS | 0.639 | — |

The HTMT value between the Perceived Stigma and Internalized Stigma dimensions was 0.639, which is well below the recommended threshold of 0.85 (Henseler et al., 2015). This indicates that, despite their high correlation, the two dimensions are distinct yet related constructs, thereby supporting the discriminant validity of the scale.

**PART 3: Priming Materials for Manipulating Perceived (Non-)Meritocratic Social Climate**

**Meritocratic Climate Priming Text:**

Imagine you are in a debate competition. You are on the affirmative side, and the affirmative position is: “We live in a social atmosphere that emphasizes achievements and values success.” Please refer to the excerpt from a recent article below as a reference for your argument. Afterwards, you need to state the article’s main points, connect them to real life, provide examples, and answer the corresponding questions.

**Arrogant Winners and Belittled Losers**

Since the reform and opening-up, China has experienced rapid development over nearly half a century, leading to tremendous increases in both national wealth and personal income. In China, everyone has an equal opportunity to succeed. Regardless of one’s starting point in life, as long as one abides by the law and works hard, one can seize opportunities and achieve a rags-to-riches transformation.

Amid the tide of national development, many people, relying on their personal abilities and efforts, have obtained higher education, found good jobs, gained relatively high social status, and accumulated personal wealth, becoming “winners” in society. Of course, there are also many who, due to bad luck, lack of ability, or insufficient effort, failed to seize opportunities for various reasons. They were unable to enter university, could not find decent jobs, have low social status, and earn meager incomes, becoming “losers” in society.

Riding the wave of the times, successful people instinctively believe that their success is entirely the result of their own abilities and efforts, overlooking factors such as policy and luck. They also believe that those losers are lowly because they are lazy, not smart enough, or incompetent, and show no compassion whatsoever for “deviants” who violate social rules and order. Meanwhile, the losers themselves also blame themselves for “not working hard enough or being incompetent,” bearing shame and blame. This constitutes the current overall social atmosphere of “rewarding winners and belittling losers.” This atmosphere of “emphasizing achievements and valuing success” spreads from society into families, schools, and workplaces. Resources and preferential treatment are tilted toward the “winners,” while the “losers” face the harsh reality of being ignored, belittled, and blamed. Whether a person has a stain or blemish on their record, their academic performance, whether they can gain admission to university, which university they attend, and what kind of job they do have become the social yardsticks that define a person as a success or a failure.

**Non-Meritocratic Climate Priming Text:**

Imagine you are in a debate competition. You are on the negative side, and the negative position is: “We live in a social atmosphere that emphasizes meaning and values experiences.” Please refer to the excerpt from a recent article below as a reference for your argument. Afterwards, you need to state the article’s main points, connect them to real life, provide examples, and answer the corresponding questions.

**Returning to Life’s Authenticity: We Are All Ordinary People**

Since the reform and opening-up, China has experienced rapid development over nearly half a century, leading to tremendous increases in both national wealth and personal income. However, as socio-economic development has entered a period of slow growth and bottlenecks, more young people in China have begun to reflect on the meaning of “success” and “failure.” They are more inclined to follow their hearts and pursue a more authentic, simple lifestyle, returning to the true essence of life.

In the generation of young people’s parents, most rode the wave of the country’s rapid development, leveraging abundant opportunities and their own efforts to become “winners” in society. They were also more accustomed to using external standards—such as grades, income, and the decency of a job—or reputation as symbols of personal success and social status, and they used these measures to evaluate their own children or other people’s children. But now, many young people of the post-00s and post-10s generations clearly realize that their parents’ success cannot be replicated. Success is not the only goal in life, because not everyone has the timing, luck, or resources needed to achieve it. Therefore, there is no need to belittle or discriminate against the so-called “losers” in society. They are more tolerant of people with different personalities, preferences, experiences, and family backgrounds. They place greater emphasis on personal happiness and the public good, rather than merely pursuing material wealth and higher social status.

Influenced by a social atmosphere that stresses meaning and experience, many parents are also re-examining the standards of “success” and affirming their children’s true worth. As one father put it, “My son is a ‘poor student,’ but I still believe he will have a bright future.” The younger generation has been exposed to more diverse values and lifestyles, and they affirm the worth of their own existence. As long as they take their lives seriously, abide by the law, and make a down-to-earth effort, even if they do not meet society’s so-called standards of success, there is no distinction between high and low, noble and humble—both the person and their work deserve respect.

**Control Group Priming Text:**

Next, we will conduct a test of imaginative ability. Please read the following article carefully, imagine the natural landscape described as vividly as possible, and tell us which description impressed you the most.

**The Grand Canyon**

‌‌ The Grand Canyon is a world-renowned natural wonder, located on the Kaibab Plateau in northwestern Arizona, USA, and is one of the most magnificent landscapes on Earth. The canyon was carved by the Colorado River over millions of years, revealing nearly two billion years of geological history.

The Grand Canyon is 2,320 kilometers long. The canyon ranges from 6 to 28 kilometers wide at the rim and reaches a maximum depth of 1,800 meters. At the bottom, the river is less than 1,000 meters wide. In summer, when snow and ice melt, the water depth increases to 18 meters. The canyon’s rocks are predominantly red; the Colorado River carries so much silt that its waters often appear red, hence the name. Grand Canyon National Park was established in 1919, covers an area of 1,904 square miles as part of the greater Grand Canyon, and was inscribed on the UNESCO World Heritage List in 1979. The park features many trails and viewpoints, such as Hermit Trail and Bright Angel Trail, allowing visitors to enjoy spectacular close-up views of the canyon. It is also home to a wealth of wildlife, including 75 mammal species, 50 amphibian and reptile species, 25 fish species, and over 300 bird species.

The best evidence of prehistoric human habitation here dates back more than 1,000 years. The Anasazi people lived in dwellings made of stone and mud. Archaeological sites dating back approximately 2,500 years have been discovered in the canyon, indicating human presence over the past 4,000 years. Anasazi ruins are scattered throughout the canyon and can be seen by visitors traveling by boat, hiking the trails, or walking along the rim.

**PART4 Dimension-Specific Correlation and Mediation Analyses of Perceived Societal Meritocratic Climate**

**Table 4.1 Correlations Between Perceived Meritocratic Climate Dimensions and Key Variables**

| Variables | 1 | 2 | 3 | 4 |
| --- | --- | --- | --- | --- |
| 1Perceived Meritocratic Evaluation Climate |  |  |  |  |
| 2Perceived Meritocratic Attribution Tendency | .729** |  |  |  |
| 3Collective self-esteem | -.132* | -.185** |  |  |
| 4Social Disconnection | .227** | .322** | -.471** |  |
| 5Delinquency-based Self-stigma | .429** | .581** | -.303** | .515** |

Note. **p* < .05, ***p* < .0 1.

**Table 4.2 Regression Results for the Serial Mediation Model: Perceived Meritocratic Climate (Evaluative) as the Independent Variable**

| Outcome variable | Predictor variable | *R* | *R^2^* | *F* | *β* | *t* |
| --- | --- | --- | --- | --- | --- | --- |
| Collective self‑esteem |  | 0.253 | 0.064 | 4.321** |  |  |
|  | Perceived meritocratic climate (Evaluative) | |  |  | -0.112 | -2.205* |
| Social Disconnection |  | 0.527 | 0.278 | 20.109*** |  |  |
|  | Perceived meritocratic climate (Evaluative) | |  |  | 0.221 | 3.259** |
|  | Collective self‑esteem |  |  |  | -0.641 | -8.614*** |
| Delinquency-based Self-stigma |  | 0.618 | 0.328 | 27.604*** |  |  |
|  | Perceived meritocratic climate (Evaluative) | |  |  | 0.427 | 7.226*** |
|  | Collective self‑esteem |  |  |  | -0.091 | -1.280 |
|  | Social Disconnection |  |  |  | 0.366 | 7.567*** |

Note. **p* < .05, ***p* < .0 1, ******p*** <.001; all models controlled for gender, age, family type, and family socioeconomic status.

**Table 4.3 Regression Results for the Serial Mediation Model: Perceived Meritocratic Climate (Attributional) as the Independent Variable**

| Outcome variable | Predictor variable | R | R2 | F | β | t |
| --- | --- | --- | --- | --- | --- | --- |
| Collective self‑esteem |  | 0.278 | 0.078 | 5.290** |  |  |
|  | Perceived meritocratic climate (Attributional) | |  |  | -0.108 | 负3.077** |
| Social Disconnection |  | 0.552 | 0.305 | 22.916*** |  |  |
|  | Perceived meritocratic climate (Attributional) | |  |  | 0.223 | 4.817** |
|  | Collective self‑esteem |  |  |  | -0.61 | -8.301*** |
| Delinquency-based Self-stigma |  | 0.683 | 0.467 | 39.158*** |  |  |
|  | Perceived meritocratic climate (Attributional) | |  |  | 0.409 | 10.515*** |
|  | Collective self‑esteem |  |  |  | -0.080 | -1.212 |
|  | Social Disconnection |  |  |  | 0.303 | 6.619*** |

Note. **p* < .05, ***p* < .0 1, ******p*** <.001; all models controlled for gender, age, family type, and family socioeconomic status.

**Table 4.3 Indirect Effects and Comparison Between the Two Dimensions (Bootstrap Analysis)**

|  | Perceived meritocratic climate (Evaluative) Model | | Perceived meritocratic climate (Attributional) Model | |
| --- | --- | --- | --- | --- |
|  | Effect (BootSE) | 95% BootCI | Effect (BootSE) | 95% BootCI |
| Tatal Indirect path decomposition | 0.118 (0.036) | [0.048, 0.191] | 0.096 (0.023) | [0.055, 0.144] |
| Ind1: X → Collective self‑esteem → Delinquency-based Self-stigma | 0.010 (0.011) | [-0.006, 0.038] | 0.009 (0.009) | [-0.007, 0.030] |
| Ind2: X → Social Disconnection → Delinquency-based Self-stigma | 0.081 (0.029) | [0.027, 0.139] | 0.068 (0.019) | [0.034, 0.107] |
| Ind3: X → Collective self‑esteem → Social Disconnection → Delinquency-based Self-stigma | 0.026 (0.014) | [0.001, 0.055] | 0.020 (0.009) | [0.005, 0.038 |

Note. Bootstrap sample size = 5,000; CI = bias-corrected percentile confidence interval.
